# Knowledge graph and bibliometric analysis of inflammatory indicators in ovarian cancer

**DOI:** 10.3389/fonc.2025.1533537

**Published:** 2025-06-30

**Authors:** Liyan Zhang, Linlin Guo, Haiyan Wang, Huan Yang, Jiarui Dong

**Affiliations:** ^1^ Department of Functional Examination, Obstetrics and Gynecology Center, General Hospital of Ningxia Medical University, Yinchuan, Ningxia, China; ^2^ Clinical Medical College, Ningxia Medical University, Yinchuan, Ningxia, China

**Keywords:** inflammatory indicators, ovarian cancer, bibliometric analysis, early diagnosis, post-treatment

## Abstract

**Background:**

Chronic inflammation is a type of inflammatory response that lasts for a relatively long period of time. Occurrence and development of many diseases are closely related to chronic inflammation. In the process of the occurrence of certain tumors, chronic inflammation also plays an important role. For example, chronic inflammation of the stomach caused by chronic Helicobacter pylori infection is an important risk factor for gastric cancer. Inflammatory cells can release reactive oxygen species (ROS), cytokines and so on which can induce DNA damage in cells, activate oncogenes and suppress tumor suppressor genes, thus promoting the proliferation, survival, migration and invasion of tumor cells. Recent studies have shown that great progress has been made in understanding the role of chronic inflammation in ovarian cancer. However, there has been no bibliometric analysis in this research field yet. The aim of this study is to review the knowledge structure and research hotspots of inflammatory indicators in ovarian cancer through bibliometric methods.

**Methods:**

A computer search was conducted on 595 articles related to inflammatory markers and ovarian cancer in the Web of Science Core Collection (WoSCC) database from 2000 to 2024. Relevant software such as VOSviewer, CiteSpace, R package “bibliometrix” and Microsoft Office Excel 2019 were used to conduct a comprehensive bibliometric analysis on these related articles and analyze the research status and development directions over the past more than 20 years.

**Results:**

A total of 595 articles related to inflammatory markers and ovarian cancer were included in this study. Among them, the United States and China had the largest number of published articles. Global publications have been steadily increasing every year with reaching a peak in 2023. The United States had the largest number of publications and followed by China and Italy. Among them, Harvard University in the United States had the largest number of published papers, approximately 100 and followed by the National Institutes of Health (NIH) and the NIH National Cancer Institute (NCI) with 52 each. CHATURVEDI AK ranked first in the total citation number of published articles, with a total of 683 citations and HILDESHEIM A ranked second in the total citation number, with a total of 651 citations. The number citations are highlighting their significant contributions to this field. The two magazines of the CANCER EPIDEMIOLOGY BIOMARKERS & PREVENTION and JOURNAL OF OVARIAN RESEARCH had the most published articles and followed by Cancer and PLOS ONE. The article “COUSSENSLU, 2002, NATURE V420, P860, 00110, 1030/NATURE01322” was the most frequently cited one, with 46 citations. The article “CRVENNKOY SL2010, CELL7140 2883, 00110 016402LL 201001025” was cited 38 times and ranked second. The analysis results of CiteSpace show that ovarian cancer, inflammation and prognosis are identified as the keywords with the highest frequencies, indicating the core research focuses and directions in this area. The results of the research topics in this area show that from 2019 to 2024, the main themes were ovarian cancer, inflammation and so on. Through the analysis of the research trends, it can be known that from 2019 to 2024, ovarian cancer and inflammation were the main trends, which is the analysis of trend topics. All in all, the indicators of ovarian cancer and inflammation represent the frontier research directions in this field.

**Conclusion:**

This is the first bibliometric study that comprehensively summarizes the research trends and progress of inflammatory indicators in ovarian cancer. The information in this study has summarized the recent research frontiers and hotspots. The results will provide references for scholars to study the early diagnosis and treatment of inflammatory indicators and ovarian cancer.

## 1 Introduction

Ovarian cancer is a kind of malignant tumor affecting the female reproductive system and usually originates from the epithelial cells of the ovary. According to the data of Global Cancer Observatory(GLOBOCAN), ovarian cancer is one of the leading causes of cancer related deaths among women which incidence rate varies significantly among different regions and populations (1). In the United States, there are significant differences in the incidence of ovarian cancer among different Asian - American women. Among them, women of Indian/Pakistani origin have the highest incidence, while Korean women have the lowest (2). Some studies have also found that compared with other types of gynecological tumors, the incidence of ovarian cancer is higher in high-income countries which may be related to lifestyle, genetic factors and the level of health care (3). In clinical practice, treatment regimens for ovarian cancer usually include multiple methods such as surgery, chemotherapy and targeted therapy. The standard chemotherapy regimen generally involves the combined use of platinum-based drugs (such as carboplatin) and paclitaxel for six cycles (4). In addition, for some patients with platinum-sensitive recurrent ovarian cancer need to treat strategies such as secondary cytoreductive surgery. Non-platinum-based chemotherapy and anti-angiogenic drugs can be considered to improve the prognosis of patients (5). However, due to the complex biological characteristics and heterogeneity of ovarian cancer, the prognosis remains poor. Therefore, the search for effective biomarkers to assist in early diagnosis and predict prognosis has become a research hotspot. In recent years, with the continuous progress of molecular biology research, the relationship between inflammatory markers and ovarian cancer has attracted extensive attention. Studies have shown that chronic inflammation may play an important role in the development of ovarian cancer. For example, some studies have found that high levels of C- reactive protein (CRP) and other inflammatory cytokines are associated with an increased risk of ovarian cancer (6). In addition, inflammatory markers in the blood, such as the neutrophil-to-lymphocyte ratio (NLR) and the platelet-to-lymphocyte ratio (PLR) are also regarded as potential prognostic indicators. These indicators can reflect the body’s response to changes in the tumor microenvironment (7, 8). Clinical studies have shown that different types of inflammatory markers are significantly associated with the survival rate of ovarian cancer patients. For example, in a large-scale prospective cohort study, high NLR and PLR values were found to be associated with a poor prognosis of ovarian cancer (9). In addition, In addition, some studies have pointed out that the density of certain specific types of infiltrating macrophages may increase the risk of ovarian cancer, which indicates the important role of the immune microenvironment in the development of the disease (10). To sum up, inflammatory markers can not only serve as an important tool for ovarian cancer risk assessment but also may provide guidance for subsequent treatment. However, at present, how these markers specifically affect the progression of ovarian cancer and its mechanism still require further in-depth research in order to better understand their value in clinical application. This study adopts the bibliometric method and focuses on quantifying the entire knowledge system of inflammatory markers for ovarian cancer. By analyzing the existing quantitative literature, intuitive maps can be used to predict the future development direction of a research field and conduct a systematic analysis of the research.

## 2 Materials and methods

### 2.1 Data collection

**Figure 1:**
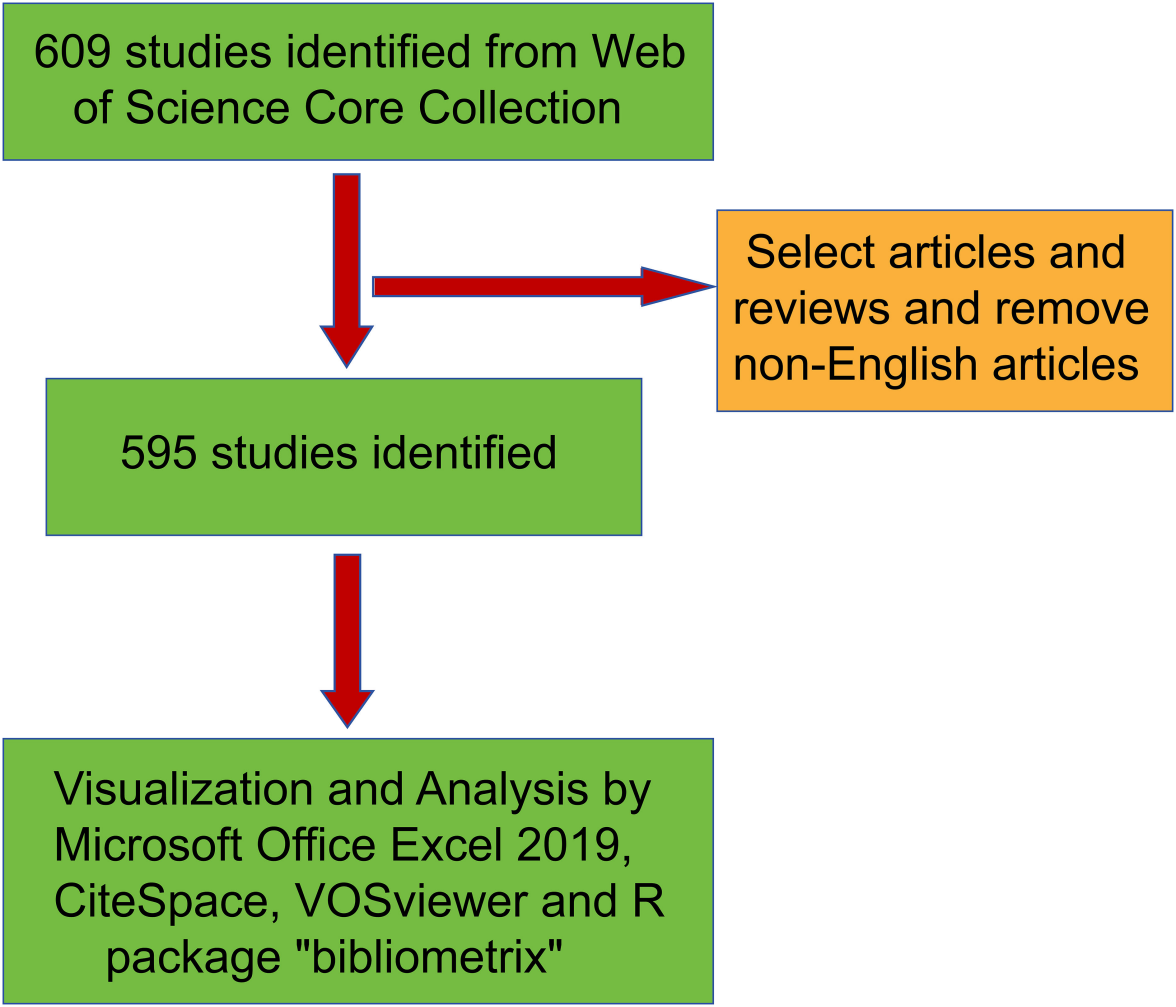
Research flow chart.

All the published papers were from WoSCC (https://www.example.com/wos/woscc/basic-search). Two researchers identified the relevant papers published during the period from January 1, 2000 to October 31, 2024. The search formula was (TS = inflammatory indicators and ovarian cancer (OR)TS = inflammatory indicator metabolism and ovarian cancer) and publication year and English. After the initial data retrieval, two researchers (LY Zhang、LL Guo) screened all the manuscripts respectively to ensure that they were relevant to the theme of this study. Any discrepancies were resolved by the experienced corresponding author (HY Wang). The document types were set as articles and review and the flowchart of this study showed in **Figure 1**. Conduct visual analysis on the records of all publications, including the year of publication, title, author’s name, affiliated institution, country/region, abstract, keywords and the name of the publishing journal. The size and color of the nodes are used to represent the quantity and classification of these items respectively.

### 2.2 Data analysis

Our bibliometric analysis followed a general-to-detailed path, including an overview of countries/regions, institutions/authors, journal distribution, documents/references, keywords and trends. The collected data were imported into Biblioshiny (R version 4.3.3), VOSviewer (version 1.6.20), CiteSpace (version 6.3.R1) and Microsoft Office Excel 2019 for further analysis. VOSviewer (version 1.6.18) is a bibliometric analysis software, which is often used to construct collaboration, co-citation and co-occurrence networks (11). In the maps created by VOSviewer, a node represents an item, such as a country, research institution, journal and author. The size and color of the node indicate the quantity and classification of these items respectively (12). In this study, this software mainly completed the analysis of countries (institutions), the analysis of journals and cocited journals, the analysis of authors and co-cited authors and the analysis of keyword cooccurrence. CiteSpace (version 6.1.R1) is another software for bibliometric analysis and visualization developed by Professor Chen C (13). In this study, CiteSpace was used to create the dual - map overlay of journals and Citation Bursts was utilized to analyze the references. The R software package “bibliometrix” (version 3.2.1) is used to conduct thematic evolution analysis and construct a network of the global distribution of publications on inflammatory markers and ovarian cancer (14). The quartiles and impact factor of the journals are from the Journal Citation Reports 2020. In addition, Microsoft Office Excel 2019 was used to conduct a quantitative analysis of the publications.

## 3 Results

### 3.1 The overall publication volume of global literature

**Figure 2:**
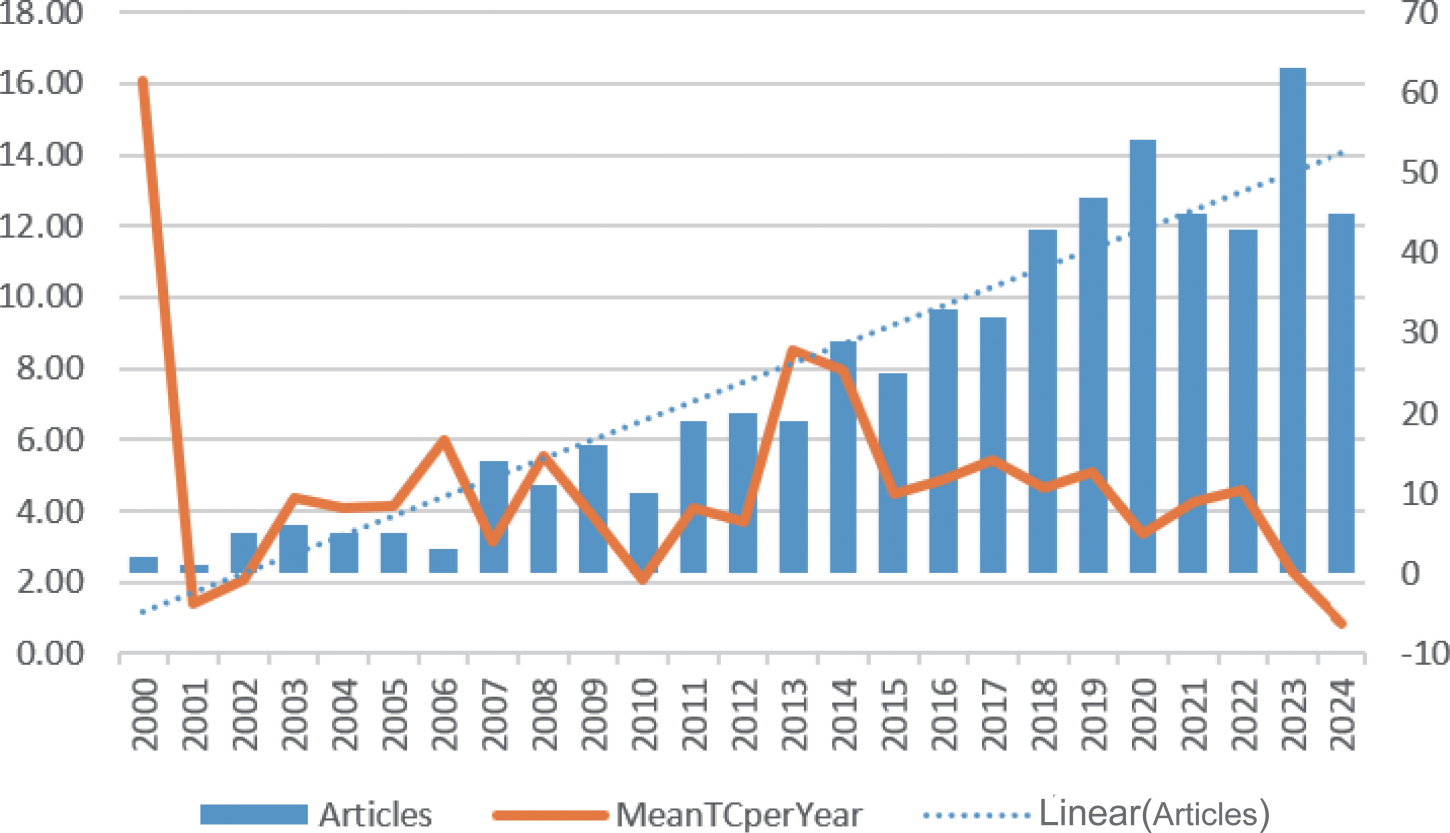
Annual output of publications and annual average articles on inflammatory markers and ovarian cancer.

According to our retrieval strategy, there were 595 studies on inflammatory markers and ovarian cancer in the past 24 years. The annual number of publications increased from 4 in 2000 to 42 in 2024, showing an upward trend, with a peak of 62 in 2023 (**Figure 2**).

### 3.2 Analysis of countries (regions)

**Figure 3:**
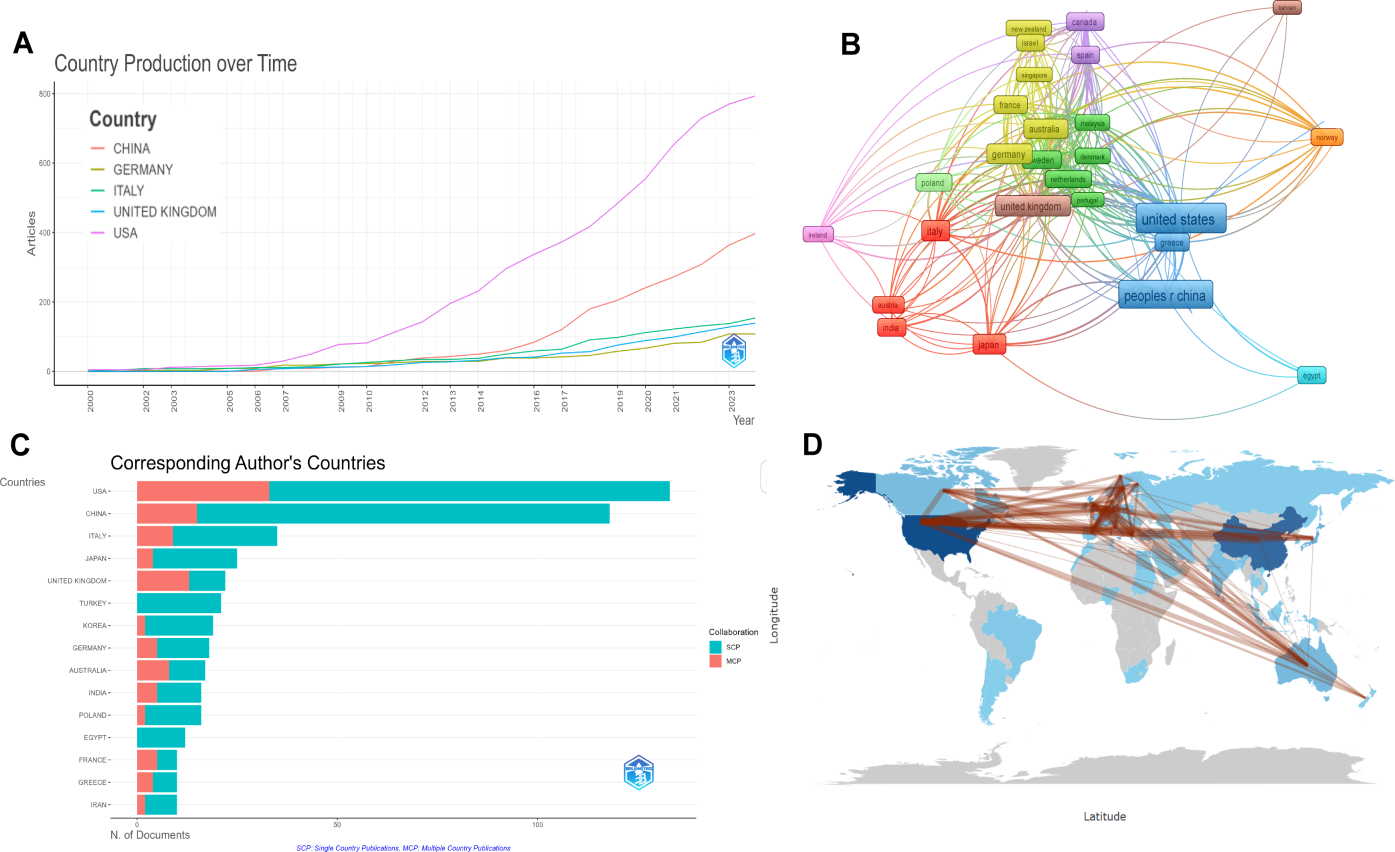
Visual analysis of the number of articles published by different countries (regions). **(A)** Changes in the number of articles published by different countries (regions) from 2000 to 2020; **(B)** Using the size of the image to represent the number of publications in different countries (regions); **(C)** Countries(regions) where the top 15 corresponding authors are located; **(D)** Visualization of countries (regions).

The number of publications in each country was analyzed to identify the countries (regions) that have made major contributions to this field. Researchers from 1,543 institutions in 58 countries have participated in the research on inflammatory markers and ovarian cancer. According to the number of published articles in different countries, the 20 countries (regions) with the largest number were analyzed. The United States ranked first, followed by China and Italy. The total number of their publications accounted for almost half of that of the other countries. The United States published 133 articles with a total of 7,366 citations, ranking first among all participating countries (regions). China published 118 articles with a total of 2,816 citations, ranking second. This indicates that the United States and China are the most important contributors in this field (**Table 1**). **Figure 3A** analyzes the data curves of the top five countries with the largest number of published papers over time. The United States is the country with the largest number of published papers, followed by China. **Figure 3B** The number of articles published by different countries and their connections and used the size of the image to represent the number of published papers. The results show that the United States and China have the largest number of published papers. **Figure 3C** Rank the countries where the top 10 authors are located according to the number of articles published by the corresponding authors. The results show that the United States has the largest number of published articles, followed by China. **Figure 3D** constructs a cooperation relationship network based on the countries where the top 15 corresponding authors are located. The results show that the United States is the country that initiates and participates in the most international cooperation, followed by China.

**Table 1:** Top 20 most productive countries/regions.

Rank	Country	Article	N%	SCP	MCP	MCP %	TC	AC
1	USA	133	22.4	100	33	24.8	7366	55.40
2	CHINA	118	19.8	103	15	12.7	2816	23.90
3	ITALY	35	5.9	26	9	25.7	1216	34.70
4	JAPAN	25	4.2	21	4	16	551	22.00
5	UNITED KINGDOM	22	3.7	9	13	59.1	1596	72.50
6	TURKEY	21	3.5	21	0	0	427	20.30
7	KOREA	19	3.2	17	2	10.5	717	37.70
8	GERMANY	18	3	13	5	27.8	708	39.30
9	AUSTRALIA	17	2.9	9	8	47.1	1139	67.00
10	INDIA	16	2.7	11	5	31.3	250	15.60
11	POLAND	16	2.7	14	2	12.5	330	20.60
12	EGYPT	12	2	12	0	0	172	14.30
13	FRANCE	10	1.7	5	5	50	1227	122.70
14	GREECE	10	1.7	6	4	40	370	37.00
15	IRAN	10	1.7	8	2	20	111	11.10
16	CANADA	9	1.5	2	7	77.8	809	89.90
17	ROMANIA	8	1.3	7	1	12.5	44	5.50
18	AUSTRIA	7	1.2	5	2	28.6	214	30.60
19	BRAZIL	6	1	6	0	0	98	16.30
20	FINLAND	6	1	2	4	66.7	180	30.00

AC, Average Citations; TC, Total Citations.

### 3.3 Analysis of universities (institutions)

**Figure 4:**
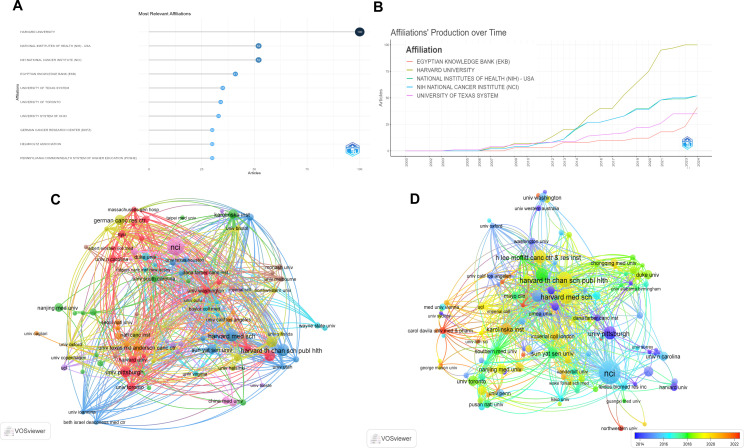
Visual analysis of universities (institutions) that published articles. **(A)** The top 10 universities (institutions) with the most published articles from 2000 to 2024; **(B)** The output volume of the top 5 universities (institutions) from 2000 to 2024; **(C)** The co-authorship map of universities (institutions) engaged in the research on inflammatory indicators and ovarian cancer from 2000 to 2024; **(D)** The co-authorship map of universities (institutions) engaged in the research on inflammatory indicators and ovarian cancer from 2014 to 2024.

Statistics were conducted on the top 15 universities (institutions) with the most published articles to identify the universities (institutions) that have made contributions in this field. The results showed that 10 universities (institutions) were from the United States and 2 universities (institutions) were from Germany. Among them, Harvard University published 100 papers, followed by the National Institutes of Health of the United States and the NIH National Cancer Institute (NCI), with 52 papers each (**Table 2**). **Figure 4A** analyzed the top 10 universities (institutions) with the largest number of published articles from 2000 to 2024. Among them, HARVARD UNIVERSITY published 100 articles with ranking first and followed by NATIONAL INSTITUTES OF HEALTH (NIH) - USA and NIH NATIONAL CANCER INSTITUTE (NCI) published 52 articles each. **Figure 4B** shows the curves of the number of articles published by the top 5 universities (institutions) over time, among which Harvard University had the fastest upward trend in changes. **Figure 4C** uses a co-authorship network diagram to display the connections among different universities (institutions) and the number of published articles. The larger the circle the more articles were published. **Figure 4D** uses a co-authorship network diagram to display the connections among different universities (institutions) and the number of published articles from 2014 to 2024. The larger the circle the more articles were published.

**Table 2:** Top 15 most productive institutions.

Rank	Affiliation	Country	Articles
1	HARVARD UNIVERSITY	USA	100
2	NATIONAL INSTITUTES OF HEALTH (NIH) - USA	USA	52
3	NIH NATIONAL CANCER INSTITUTE (NCI)	USA	52
4	EGYPTIAN KNOWLEDGE BANK (EKB)	Egypt	41
5	UNIVERSITY OF TEXAS SYSTEM	USA	35
6	UNIVERSITY OF TORONTO	Canada	34
7	UNIVERSITY SYSTEM OF OHIO	USA	33
8	GERMAN CANCER RESEARCH CENTER (DKFZ)	Germany	30
9	HELMHOLTZ ASSOCIATION PENNSYLVANIA COMMONWEALTH	Germany	30
10	SYSTEM OF HIGHER EDUCATION (PCSHE)	USA	30
11	UNIVERSITY OF PITTSBURGH	USA	29
12	DUKE UNIVERSITY	USA	27
13	HARVARD MEDICAL SCHOOL	USA	26
14	UNIVERSITY OF LONDON	England	26
15	UTMD ANDERSON CANCER CENTER	USA	25

### 3.4 Authors and co-authors

**Figure 5:**
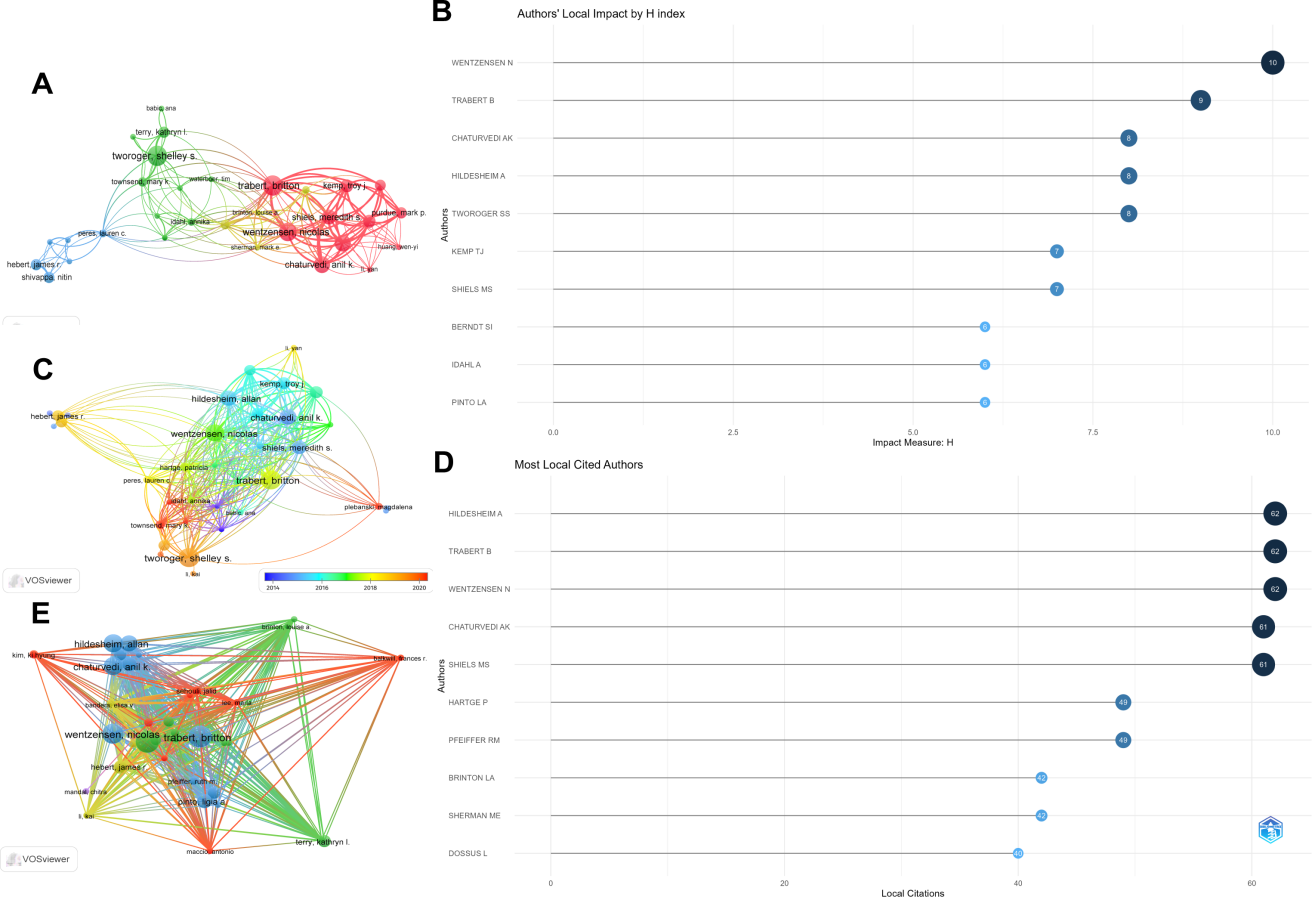
Visual analysis of authors and co-authors. **(A)** The connections between authors and cocited authors; **(B)** The influence of the H-index of local authors; **(C)** The connections between authors and co-cited authors from 2014 to 2020; **(D)** The local authors with the most citations; **(E)** The mutual connections among authors in the research on inflammatory indicators and ovarian cancer.

Conduct statistical research on the authors who have published works on inflammatory indicators and ovarian cancer and sort them according to the H-index. The results show that the article published by WENTZENSEN N has the highest H-index, followed by TRABERT B. When sorted according to the total number of citations, the results show that CHATURVEDI AK ranks first with 683 citations and HILDESHEIM A ranks second with 651 citations (**Table 3**). **Figure 5A** constructs a collaboration network based on the number of papers published. Among them, WENTZENSEN N and TRABERT B have the largest nodes indicating that they have published the most articles. Therefore, we closely observed the cooperation among authors. **Figure 5B** represents the ranking of this region according to the level of the H-index and among which WENTZENSEN N ranked first, followed by TRABERT B. **Figure 5C** constructs a collaboration network based on the number of papers published by different authors from 2014 to 2020. Among them, the node of WENTZENSEN N is the largest, indicating that the number of published papers is the largest. **Figure 5D** shows the authors with the most citations of local and among which HILDESHEIM A ranked first, followed by TRABERT B. **Figure 5E** uses the author coupling method to show the connections among different authors.

**Table 3:** The top 15 authors with the most published articles.

Rank	Author	H index	G index	M index	TC
1	WENTZENSEN N	10	11	0.909	625
2	TRABERT B	9	11	0.818	578
3	CHATURVEDI AK	8	9	0.667	683
4	HILDESHEIM A	8	9	0.667	651
5	TWOROGER SS	8	11	0.8	187
6	KEMP TJ	7	7	0.583	575
7	SHIELS MS	7	8	0.583	636
8	BERNDT SI	6	6	0.462	440
9	IDAHL A	6	6	0.429	232
10	PINTO LA	6	7	0.5	526
11	WANG Y	6	8	0.5	92
12	DOSSUS L	5	5	0.313	234
13	FORTNER RT	5	6	0.5	165
14	HARTGE P	5	5	0.455	248
15	KATKI HA	5	6	0.417	511

TC, Total Citations.

### 3.5 Journal distribution

**Figure 6:**
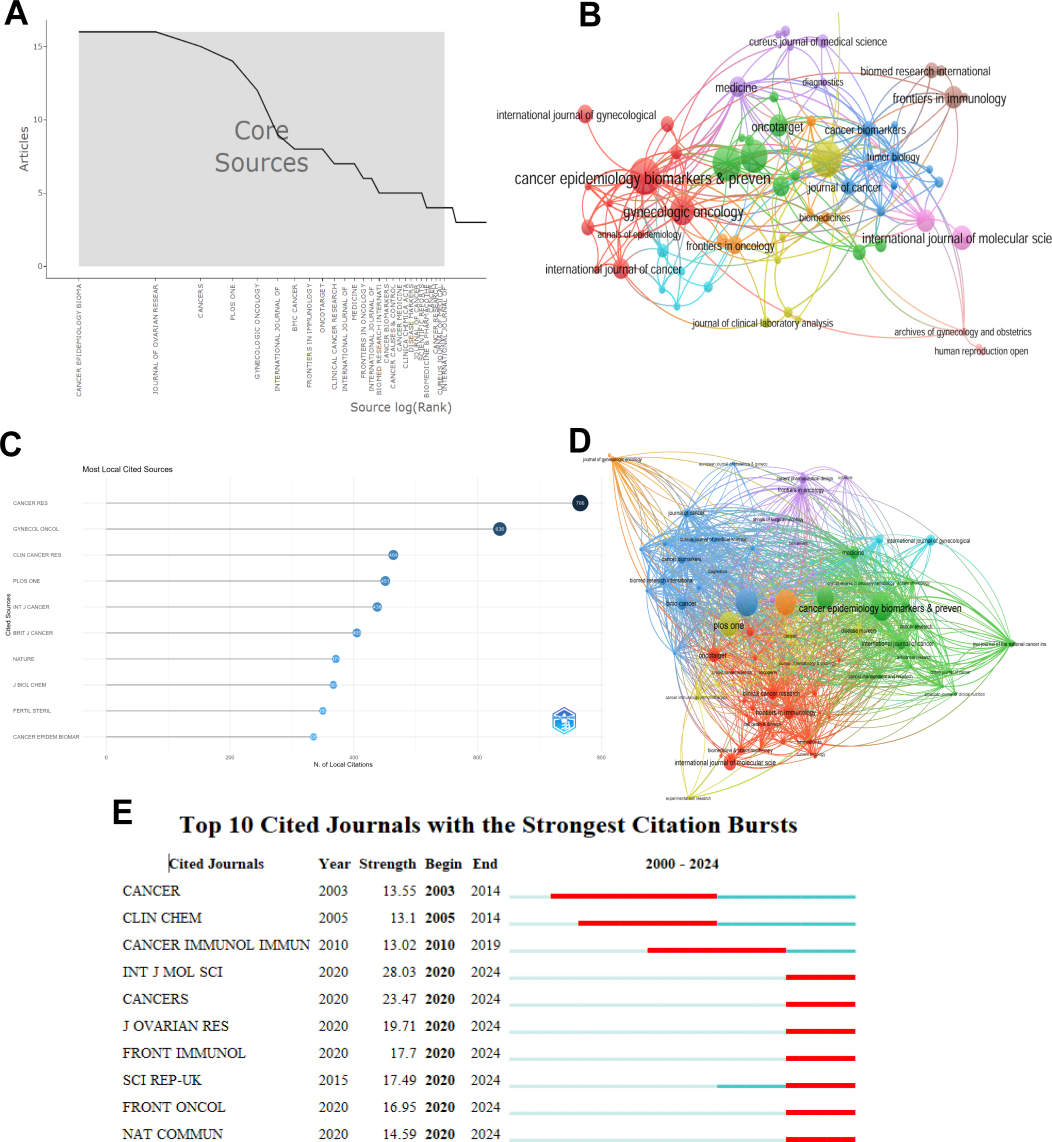
Visual analysis of the number of publications in different journals. **(A)** Changes in the number of core journals related to inflammatory indicators of ovarian cancer; **(B)** The number of publications in different journals; **(C)** The top 10 journals with the most local citations; **(D)** Connections between different journals; **(E)** The top 10 journals with relatively strong citation rates.

Conduct statistical research on the journals that have published articles on inflammatory indicators and ovarian cancer. Fifteen journals with the most published articles were collected. Among them, the two magazines, namely CANCER EPIDEMIOLOGY BIOMARKERS & PREVENTION and JOURNAL OF OVARIAN RESEARCH published the most articles, followed by Cancer and PLOS ONE (**Table 4**). **Figure 6A** shows the changes in the number of core journals related to inflammatory indicators of ovarian cancer. Among them, CANCER EPIDEMIOLOGY BIOMARKERS &

**Table 4:** The most relevant journals.

Rank	Sources	IF	Articles	TC
1	PREVENTION	3.7	16	335
2	JOURNAL OF OVARIAN RESEARCH	4.4	16	133
3	CANCERS	5.6	15	145
4	PLOS ONE	3.7	14	451
5	GYNECOLOGIC ONCOLOGY INTERNATIONAL JOURNAL OF MOLECULAR	4.4	12	636
6	SCIENCES	6.2	9	173
7	BMC CANCER	4.1	8	198
8	FRONTIERS IN IMMUNOLOGY	7.4	8	161
9	ONCOTARGET	2.4	8	268
10	CLINICAL CANCER RESEARCH	11.8	7	45
11	INTERNATIONAL JOURNAL OF CANCER	6.5	7	438
12	MEDICINE	1.1	7	82
13	FRONTIERS IN ONCOLOGY INTERNATIONAL JOURNAL OF GYNECOLOGICAL	4.5	6	99
14	CANCER	3.0	6	1
15	BIOMED RESEARCH INTERNATIONAL	3.1	5	62

TC, Total Citations.

PREVENTION and JOURNAL OF OVARIAN RESEARCH published the most articles. **Figure 6B** uses a co-authorship network diagram to represent the number of articles published by different journals and their connections. The size of the circles represents the number of articles published by different journals. **Figure 6C** shows the 10 magazines with the most local citations. Among them, CANCER RES has been cited 766 times and ranks first, while GYNECOL ONCOL has been cited 636 times and ranks second. **Figure 6D** uses a co-authorship network diagram to indicate that cancer epidemiology biomarkers & prevention has a positive co-citation relationship with JOURNAL OF OVARIAN RESEARCH, CANCERS and PLOS ONE. The dual-map overlay of journals shows the citation relationships between journals and co-cited journals. The left side is the cluster of citing journals and the right side is the cluster of cited journals. **Figure 6E** The top 10 journals with the highest citation intensity from 2003 to 2020. The length of the red bars indicates the strength of the citation rate as well as its duration.

### 3.6 Literature citation and co-citation of references

**Figure 7:**
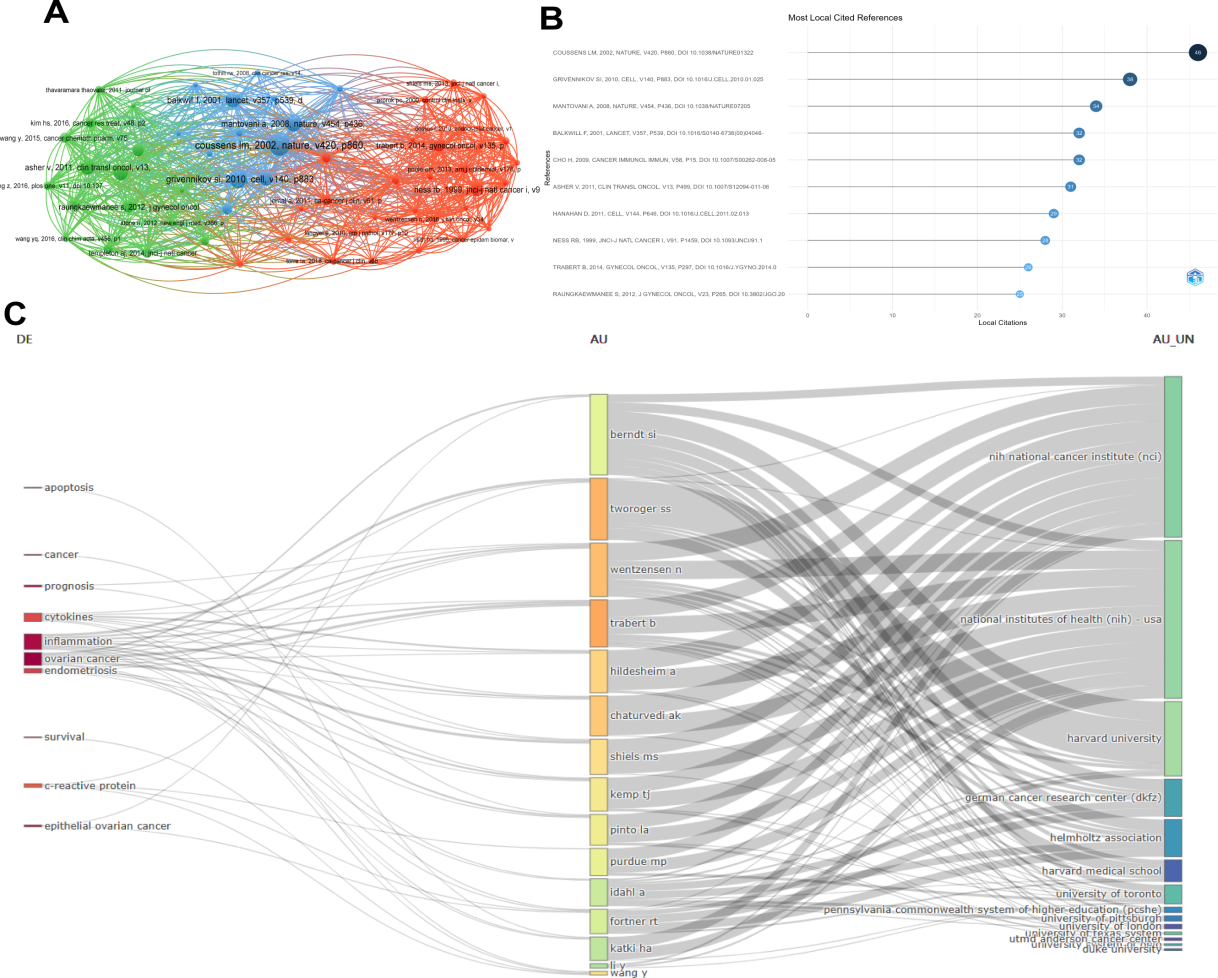
Visual analysis of literature citation and co-citation of references. **(A)** Co-authorship map of the citation and co-citation frequencies of different references; **(B)** The top 10 references with the most local citations; **(C)** Three-field diagram of high-frequency words - authors - institutions.

**Figure 7A** Statistics on the citations of references were conducted based on the citation data of papers included in the Web of Science Core Collection (WoSCC). COUSSENSLU, 2002 NATURE V420, P860, 00110, 1030/NATURE01322 has a positive co-citation relationship with GRIVENNIKOV SI. 2010. CELL. V140, P883, MANTOVANIA. 2008. NATURE, V454. P436 etc.

The dual-map overlay of the literature shows the citation relationships among the literature. The left side is the cluster of citing literature and the right side is the cluster of cited literature. **Figure 7B** The top 10 publications with the most local citations. Among them, COUSSENSLU, 2002 NATURE V420, P860, 00110, 1030/NATURE01322 ranked first with 46 citations and GRIVENNIKOV SI. 2010. CELL. V140, P883 ranked second with 38 citations. **Figure 7C** It is a three-field diagram of high-frequency words-authors-institutions. The diagram shows that ovarian cancer and inflammation are the most common words. Harvard University and the National Institutes of Health (NIH) of the United States are the institutions that have published the most articles, and WENTZENSEN N and TRABERT B are the authors who have published the most articles.

### 3.7 Keyword and research trend analysis

**Figure 8:**
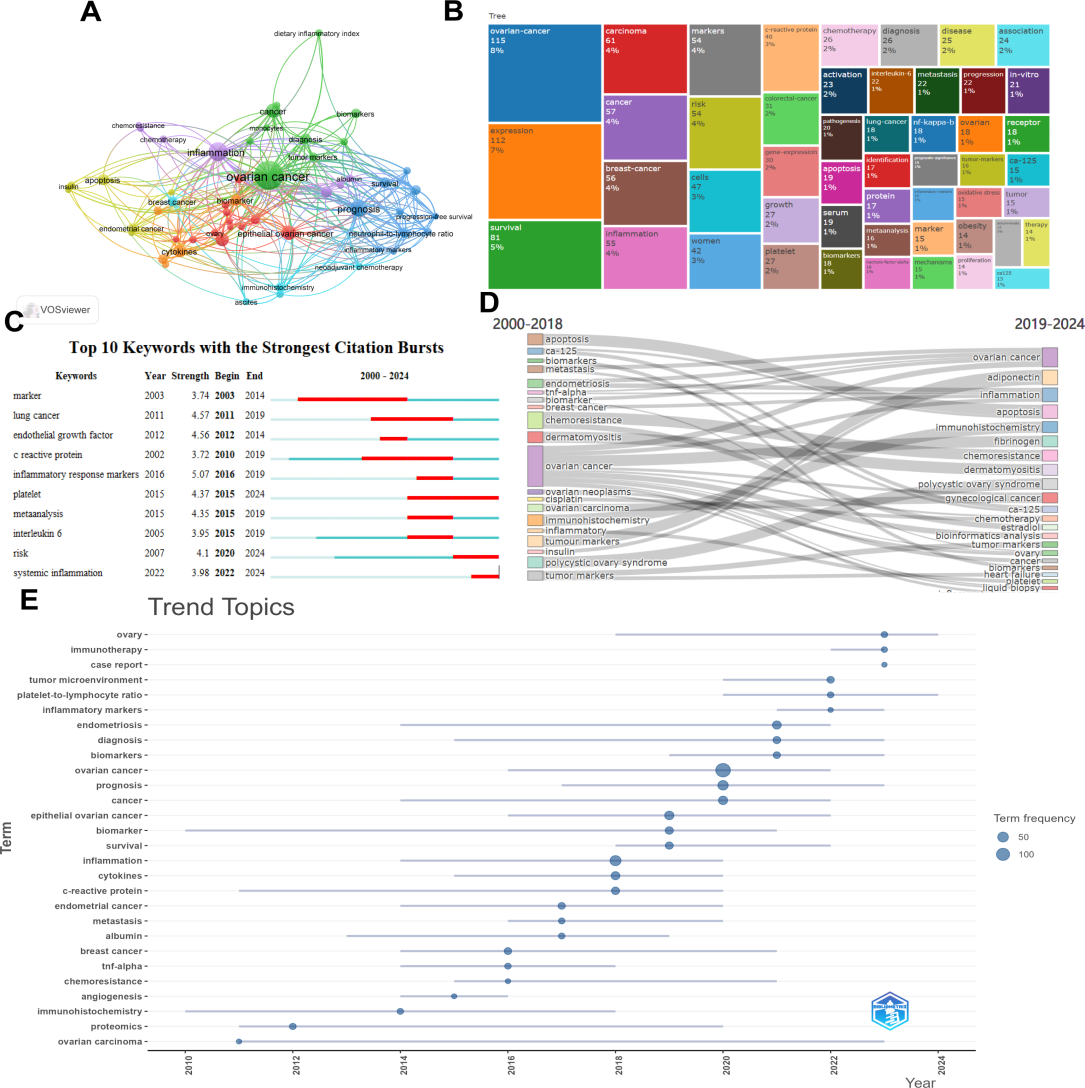
Visual analysis of keywords. **(A)** Keyword network map; **(B)** Dendrogram of the association between inflammatory indicators and ovarian cancer; **(C)** The top 10 keywords with the strongest citation bursts; **(D)** Comparison of research themes between 2000–2018 and 2019 – 2024; **(E)** Trend analysis of the research on inflammatory indicators and ovarian cancer.

**Figure 8A** uses CiteSpace to analyze the keywords of articles. The frequency of keyword occurrence is represented by the size of the circles. Among them, ovarian cancer, inflammation and prognosis have the highest occurrence frequencies. This result summarizes the directions and focuses of this research field. **Figure 8B** is a dendrogram of the association between inflammatory indicators and ovarian cancer. The main research directions are represented by the proportion of rectangles in the entire dendrogram. The results show that ovarian cancer, expression, survival and inflammation are the main research directions in this field. **Figure 8C** displays the top 10 keywords with the most citations, aiming to reflect the research hotspots and trends. **Figure 8D** The comparison between the research themes from 2000 to 2018 and from 2019 to 2024 is presented. The results show that the themes from 2000 to 2018 were mainly about ovarian cancer, chemoresistance etc. while the themes from 2019 to 2024 were mainly about ovarian cancer and inflammation etc. **Figure 8E** Trend analysis of the research on inflammatory indicators and ovarian cancer. The main trends of the research from 2000 to 2018 were C-reactive protein, endometrial cancer, metastasis, chemoresistance etc. The main trends of the research from 2018 to 2024 were inflammation, cytokines, survival, biomarkers, prognosis, immunotherapy etc. However, the frequencies of ovarian cancer, inflammation and prognosis were relatively high.

## 4 Discussion

Chronic inflammation is increasingly recognized as a key factor in the development and progression of various malignant tumors, including ovarian cancer. The inflammatory microenvironment contributes to the proliferation, survival, migration and invasion of tumor cells, as inflammatory cells such as macrophages and neutrophils release excessive inflammatory mediators, including reactive oxygen species (ROS), cytokines and chemokines. These mediators can induce DNA damage, activate proliferative signaling pathways and promote angiogenesis thus supporting tumor growth. In the context of ovarian cancer, chronic inflammatory diseases, such as pelvic inflammatory disease may be potential risk factors because inflammatory cells and mediators can alter the ovarian microenvironment and lead to abnormal proliferation and malignant transformation of ovarian epithelial cells. Due to the unobvious early symptoms and the limitations of current diagnostic methods, the early detection of ovarian cancer remains challenging which highlights the need for novel biomarkers, especially inflammatory markers which have shown promise in identifying ovarian cancer at an early stage compared with benign ovarian diseases (15, 16).

Ovarian cancer is a heterogeneous disease with diverse subtypes that pose significant challenges in diagnosis, treatment, and prognosis (17). The levels of CRP and IL - 6 in the serum of patients with ovarian cancer are higher than those in healthy people and patients with benign ovarian diseases. The results showed that inflammatory indicators have certain value for the early diagnosis of ovarian cancer (18). Recent research has increasingly focused on the role of inflammatory markers as potential biomarkers because these markers can reflect the underlying pathophysiological processes related to tumor progression and response to chemotherapy (19, 20). In this study, a bibliometric analysis of the literature on inflammatory markers of ovarian cancer from 2000 to 2024 was conducted using the Web of Science Core Collection database. By using advanced bibliometric tools such as VOSviewer, CiteSpace and the “bibliomeography” R package, the trends and contributions of various countries of researchers in this field were analyzed. The research results showed that the United States and China are the major contributors. With the development of molecular biology, more and more scholars have focused on the connection between the changes of inflammatory markers and ovarian cancer. They have also analyzed and emphasized the importance of inflammatory markers in predicting clinical outcomes and treatment responses and highlighted their potential utility in enhancing personalized treatment strategies for ovarian cancer patients (21, 22).

In this study, Renowned institutions such as Harvard University and National Institutes of Health were identified as major contributors in this area. The analysis emphasized that ovarian cancer, inflammation and prognosis were the most frequently used keywords with reflecting the core focus of current research. It is worth noting that since 2019, the emergence of the term inflammatory response markers has highlighted a shift toward exploring the role of inflammation in the prognosis and treatment of ovarian cancer. This study aims to emphasize the importance of inflammatory markers as potential biomarkers for the early diagnosis and prognosis of ovarian cancer with paving the way for future research in this important field of oncology (23, 24).

In recent years, the relationship between chronic inflammation and ovarian cancer has received high attention. Research findings show that compared with the healthy control group, the levels of C-reactive protein (CRP) and interleukin-6 (IL-6) in patients with ovarian cancer are elevated. Therefore, it is speculated that they can be used as potential indicators for the early detection of ovarian cancer (25). Monitoring inflammatory indicators can provide a non-invasive method to identify high-risk groups, thus facilitating timely intervention. This study provided a basis for the connection between inflammatory markers and ovarian cancer and fills an important gap in the existing literature. Previous studies have already determined the role of inflammation in various types of cancer (26). In this study, the use of bibliometric software analysis highlights the inflammatory indicators related to ovarian cancer. In addition, our research results support a new consensus that inflammation indices, such as the systemic inflammatory response index (SIRI) are prognostic markers for patients with ovarian cancer (27).

C-reactive protein (CRP) is an acute-phase protein synthesized by the liver during inflammatory responses. When the body is infected, traumatized, or inflamed, CRP levels rise rapidly. In ovarian cancer patients, an inflammatory response occurs, leading to elevated CRP levels. Studies have shown that serum CRP levels in ovarian cancer patients are significantly higher than those in healthy individuals, making it a useful auxiliary indicator for ovarian cancer diagnosis (28, 29).

Interleukin-6 (IL-6) is a pleiotropic cytokine that plays a key role in immunoregulation, inflammatory responses, and hematopoiesis. Ovarian cancer cells can secrete IL-6, and serum and ascites IL-6 levels are significantly elevated in ovarian cancer patients. Research has found that IL6 levels are associated with the stage and grade of ovarian cancer, providing a certain reference value for its diagnosis (30, 31).

The neutrophil-to-lymphocyte ratio (NLR) is an indicator reflecting the body’s inflammatory response and immune status, calculated by the ratio of neutrophils to lymphocytes in peripheral blood. In ovarian cancer patients, NLR elevation typically occurs due to chronic inflammatory responses induced by the tumor. Studies show that NLR in ovarian cancer patients is significantly higher than in healthy controls and is associated with ovarian cancer staging, making it a potential diagnostic indicator. However, specificity issues exist, and comprehensive diagnosis requires combination with other indicators (32, 33).

Additionally, bibliometric methods can identify emerging topics with potential but insufficient attention by analyzing keyword co-occurrences in literature and topic models. For example, in ovarian cancer research, emerging metabolic pathways or biomarkers related to inflammatory indicators may be discovered. These topics might be overlooked in traditional literature reviews due to the lack of systematic quantitative analysis. Bibliometric analysis can excavate these hidden emerging themes from large datasets, providing new research directions for investigators.

The results of this study can not only provide reliable diagnostic and treatment information for clinical practice but also guide the formulation of oncology policies. It emphasizes the importance of inflammatory indicators in routine diagnosis and treatment regimens and therapies, especially in customizing immunotherapy and chemotherapy regimens. The results of this study show that the application of inflammatory indicators may improve patient management strategies and ultimately contribute to improving the survival outcomes of patients with ovarian cancer. Such a shift in clinical practice may significantly change the efficacy of ovarian cancer treatment and the prognosis of patients. as evidenced by the positive correlation between lymphocyte levels and overall survival in patients treated with bevacizumab (34, 35).

The dominance of keywords such as “ovarian cancer”, “inflammation”, and “prognosis” indicates that one of the current research priorities in ovarian cancer is to deeply explore the pathogenesis and disease progression of ovarian cancer, particularly the role of inflammation in this process. By revealing these mechanisms, it helps to identify new diagnostic markers and therapeutic targets, providing a theoretical basis for the early diagnosis and precision treatment of ovarian cancer.

The focus on “prognosis” reflects the research characteristic of emphasizing clinical applications. That is, by studying the factors affecting prognosis, an effective prognostic evaluation system can be established, and the achievements of basic research can be translated into clinical practice to improve the treatment outcomes and quality of life of ovarian cancer patients. This also reflects the important position of translational medicine in ovarian cancer research, emphasizing the close combination of basic research and clinical practice. Finally, bibliometric methods can discover emerging topics that have not been widely concerned but have potential by analyzing the cooccurrence of keywords in literature and topic models. For example, in the research on ovarian cancer, some emerging metabolic pathways or biomarkers related to inflammatory indicators may be found. Due to the lack of systematic quantitative analysis, these topics may be ignored in traditional literature reviews. Bibliometric analysis can excavate these hidden emerging topics from a large number of literatures, providing new research directions for researchers.

This study is not without limitations. The analysis and research on bibliometric data is a retrospective study and cannot truly cover all relevant or emerging research trends. In addition, reliance on the Web of Science Core Collection database may introduce biases, as this database does not include all relevant literature and may exclude important studies published in other databases or journals. The time range from 2000 to 2024 might overlook earlier pioneering work that laid the foundation for the current research on inflammatory markers in ovarian cancer. Finally, this study only included articles in the English language and had certain limitations in the scope of inclusion. Future studies should include research subjects with different clinical backgrounds, geographical locations, languages etc. The research can include more extensively verify the role of inflammatory markers in the early diagnosis and prognosis of ovarian cancer by combining multi-center longitudinal studies as much as possible. Longitudinal studies based on multi-center data will improve the reliability of research results and be conducive to formulating guidelines that take changes in inflammatory indicators as a reference for the early diagnosis and prognostic treatment of ovarian cancer globally.

## 5 Conclusion

Inflammatory indicators have important research value and application prospects in ovarian cancer. In recent years, with the continuous increase in research literature, the research on inflammatory indicators in ovarian cancer has attracted more and more attention from scholars all over the world. In particular, the publication rates from leading institutions in the United States and China have emphasized the importance of this research in the early diagnosis and prognosis of ovarian cancer. However, the cooperation and exchanges among countries and institutions still need to be strengthened. On the one hand, studying the mechanism of the role of changes in inflammatory indicators in the occurrence and development of ovarian cancer helps to analyze the causes of the imbalance of inflammatory indicators and is beneficial for the early diagnosis of ovarian cancer and understanding the pathogenesis process. On the other hand, the treatment of ovarian cancer is the focus of research for clinicians. Changes in inflammatory indicators have great advantages in the treatment of ovarian cancer. The changes in inflammatory indicators will have great application value for the precise treatment of ovarian cancer in the future. It is worth noting that in addition to basic research, we should also pay attention to the transformation of research achievements, that is, the clinical application of changes in inflammatory indicators in patients with ovarian cancer.

## Data Availability

The original contributions presented in the study are included in the article/supplementary material, further inquiries can be directed to the corresponding author/s.
